# Antitumor effect of Kanglaite® injection in human pancreatic cancer xenografts

**DOI:** 10.1186/1472-6882-14-228

**Published:** 2014-07-08

**Authors:** Ying Liu, Wei Zhang, Xiao-Jie Wang, Shi Liu

**Affiliations:** 1 Department of Oncology, The Third Affiliated Hospital of Qiqihar Medical University, 27 Taishun Road, Qiqihar, China; 2 Heilongjiang Institute of Dermatology and Sexually Transmitted Disease, 30 Mujie Road, Harbin, China; 3 Department of General Surgery, The Third Affiliated Hospital of Qiqihar Medical University, 27 Taishun Road, Qiqihar 161099, China

**Keywords:** Kanglaite® injection, Pancreatic cancer, PI3K/Akt/mTOR signaling, Traditional Chinese medicine

## Abstract

**Background:**

Kanglaite® injection (KLT), with a main ingredient of *Coix* seed oil (a traditional Chinese medicine), has been widely used for cancer treatment in China. KLT has an inhibitory effect on many kinds of tumors and PI3K/Akt/mTOR signaling promotes cell survival, proliferation, and progression in cancer cells. Therefore, targeting this pathway may lead to the development of novel therapeutic approaches for human cancers.

**Methods:**

Here, we examined the effects of KLT on the PI3K/Akt/mTOR pathway in pancreatic cancer xenografts in mice, and assessed its therapeutic potential. Growth and apoptosis of tumor xenografts were examined, and the expression levels of genes and proteins involved in the PI3K/Akt/mTOR pathway were measured by RT-PCR and western blotting, respectively.

**Results:**

Our results revealed that KLT dramatically inhibited the growth of pancreatic cancer xenografts and induced apoptosis simultaneously. Furthermore, it downregulated the expression of phospho-Akt and phospho-mTOR.

**Conclusions:**

These results suggest that KLT can suppress growth and induce apoptosis of pancreatic cancer xenografts. Moreover, KLT can downregulate the expression of phospho-Akt and phospho-mTOR to modulate the PI3K/Akt/mTOR signaling pathway.

## Background

Pancreatic cancer is one of the most aggressive and treatment-refractory cancers, and its 5-year survival rate is less than 5% [1]. Therefore, effective new medicines are urgently required to achieve greater survival of pancreatic cancer patients. In recent years, traditional Chinese medicine has been widely used in China as adjuvant treatment during chemo- and radiotherapy for cancer [2]. *Coix* seed is a Chinese herbal medicine that is used in the treatment of hypertension, arthritis, cancer metastasis, asthma, and immunological disorders [3]. The main ingredient of Kanglaite® injection (KLT) is *Coix* seed oil [4], and it has been widely used for cancer treatment in China [5].

Preclinical studies have found that KLT may block the tumor cell cycle at the G2/M phase, and induce tumor cell apoptosis by up-regulating the expression of Fas/Apo-1 and down-regulating the expression of Bcl-2 and COX-2 [2-8]. KLT is mainly used for the treatment of non-small-cell lung cancer, liver cancer, and gastric cancer [2,5,9]. KLT has been found to significantly decrease cancer cachexy, improve quality of life of cancer patients, and may ameliorate multiple drug resistance of cancers when combined with radiotherapy and chemotherapy in clinical use [3,4].

Molecular targeting therapy against defined signaling pathways has recently attracted much attention. PI3K/Akt/mTOR is a pro-survival signaling pathway, and regulates cellular functions such as proliferation, differentiation, and migration [10]. Dysregulation of this pathway has been implicated in several cancers [11], including human pancreatic cancer. Activated PI3K/Akt/mTOR signaling impedes tumor cell apoptosis, promotes proliferation, angiogenesis, invasion, and metastasis [12].

While KLT alone cannot cure lethal human malignancies such as pancreatic cancer, there are no previous findings on the effect of KLT on the PI3K/Akt/mTOR pathway in treating pancreatic cancer. Therefore, we studied the anti-cancer effect of KLT on the PI3K/Akt/mTOR pathway in treating pancreatic cancer. In the present study, nude mice with human pancreatic cancer were treated with different concentrations of KLT and the resulting changes in the tumor growth inhibition rate, apoptosis, and protein expression levels of PI3K/Akt/mTOR pathway components were measured to explore the therapeutic mechanism of KLT in pancreatic cancer.

## Methods

### Cell culture

The human pancreatic cancer cell line PANC-1 was obtained from the Shanghai Institute of Biochemistry and Cell Biology, Chinese Academy of Sciences (Shanghai, China). Cells were cultured at 37°C in a 5% CO_2_ and 95% air humidified atmosphere in RPMI 1640 (Gibco, USA) supplemented with 10% fetal bovine serum (Hyclone, USA) and 1% penicillin-streptomycin.

### Animals and pancreatic cancer model

Male BALB/c nude mice aged 6–8 weeks (weighing 18–22 g) were purchased from the Shanghai Experimental Animal Center (Shanghai, China) and KLT was purchased from Zhejiang Kanglaite Pharmaceutical Co., Ltd (Hangzhou, China). The mice were kept in autoclaved cages with polyester fiber filters to avoid contact with the pathogens in a 12-h light/12-h dark cycle at 22–25°C and a humidity of 50–70%. Mice were fed with standard laboratory food and water. A subcutaneous injection of 5 × 10^6^ pancreatic cancer PANC-1 cells suspended in 0.1 mL phosphate buffered saline (PBS) was administered in the right flank of each mouse, allowing for the development of subcutaneous tumors after 10 days. Following tumor formation, mice (n = 40) were randomly divided into four groups of 10 each: a control group; and KLT 6.25, 12.5, and 25 mL/kg groups. Starting on the 11th day, mice in the KLT-treated groups were injected daily with KLT intraperitoneally for 21 days. Mice in the control group were injected with 0.9% normal saline. The body weights of mice and tumor volumes were also measured every 3 days. Tumor volumes were calculated with the formula: tumor volume (mm^3^) = maximal length × maximal width^2^/2 [13]. Mortality was also monitored daily. After harvesting, tumors were weighed and the tumor growth inhibition rate was calculated using the formula: (mean tumor weight of control group − mean tumor weight of treatment group)/mean tumor weight of control group × 100% [14]. The 40 tumors from the four groups were sliced into three parts for examination. All animal experiments were approved by the Animal Use and Management Committee of Qiqihar Medical University.

### TUNEL assays

Terminal deoxynucleotidyl transferase-mediated dUTP nick end labeling (TUNEL) assay was used to study DNA fragmentation using the *in situ* cell death detection kit (Roche Diagnostics, Germany) according to the manufacturer’s instructions [15]. Briefly, 40 xenograft tumor slices were fixed in 4% paraformaldehyde, embedded in paraffin, and cut into 6-μm sections. Sections of tumor tissue were treated for 20 min with Proteinase K (20 μg/mL in PBS), equilibrated for 10 min with TdT buffer, and incubated for 2 h in the TdT mix containing 100 U TdT and 0.5 μL Biotin-16-dUTP. Biotin-16-dUTP labeled in 3-OH of DNA was detected by streptavidin-HRP and visualized using diaminobenzidine for color reaction according to the manufacturer’s instructions. TUNEL-positive cells were counted in 10 randomly selected 400× high-power fields under an Olympus microscope (Olympus Inc., Japan). The apoptotic index was calculated as: the number of apoptotic cells/total number of nucleated cells × 100%.

### RT-PCR analysis

Human PI3K, Akt, and mTOR mRNA expression was evaluated by RT-PCR as described previously [16]. GAPDH served as the internal control. In brief, 40 tumor specimens (100–150 mg each) from each group were washed in PBS and minced into small pieces using bistouries. Then, samples were suspended in 1 mL of cold homogenizing buffer and homogenized in an ice-cold grinder. Total RNA was isolated from homogenate using TRIzol (Invitrogen, USA). RT-PCR was performed using the AccessQuick RT-PCR System (Promega, USA) according to the manufacturer’s instructions. The primer sets were as follows: PI3K, 5′-AGG AGC GGT ACA GCA AAG AA-3′ and 5′-GCC GAA CAC CTT TTT GAG TC-3′; Akt, 5′-TGA AAA CCT TCT GTG GGA CC-3′ and 5′-TGG TCC TGG TTG TAG AAG GG-3′; mTOR, 5′-CTG GGA CTC AAA TGT GTG CAG TTC-3′ and 5′-GAA CAA TAG GGT GAA TGA TCC GGG-3′; GAPDH, 5′-GGA AGG TGA AGG TCG GAG T-3′; and 5′-CCT GGA AGA TGG TGA TGG G-3′. Thirty cycles of amplification were performed under the following conditions: denaturation at 94°C for 30 s, annealing at 58°C (59°C for PI3K and 56°C for mTOR) for 30 s, and extension at 72°C for 1 min. The PCR products were analyzed by electrophoresis on a 2% agarose gel, and image analysis was performed using NIH Image software (Scion, USA). The experiments were repeated three times.

### Western blotting

Protein extraction and western blotting analysis of tumor cells were performed as described previously [14]. Briefly, 40 tumor specimens (100–150 mg each) from each group were washed in PBS and minced into small pieces using bistouries. Tissue samples were suspended in 1 mL of cold homogenizing buffer and homogenized in an ice-cold grinder. Then, the homogenate was centrifuged at 12,000 *g* for 15 min at 4°C. The supernatants were used immediately or stored at −20°C. Then, the protein concentrations were determined using a bicinchoninic acid protein assay kit (Pierce, USA). Equal amount of protein was separated by 15% SDS-PAGE at 80 V for 1.5 h and transferred onto polyvinylidene difluoride membranes (Millipore, USA) at 100 V for 2.5 h. After incubation in blocking solution (PBS with 10% non-fat dry milk and 0.05% Tween-20) for 1 h at room temperature, the membranes were incubated with a primary antibody (rabbit anti-PI3K, rabbit anti-Akt, mouse anti-pAkt, rabbit anti-mTOR, rabbit anti-p-mTOR, mouse anti-β-actin; Cell Signaling, USA) at a dilution of 1:1000 in TBS-Tween-20 (TBST; Sigma, USA) overnight at 4°C. Membranes were then incubated with secondary antibodies (1:2000 dilution in TBST; Cell Signaling) conjugated with horseradish peroxidase. After washing, the blots were developed using an enhanced chemiluminescence detection system (Amersham International, UK) according to the manufacturer’s protocol. Relative protein levels were corrected by employing β-actin as the internal standard. Experiments were repeated three times.

### Statistical analysis

Statistical analysis was performed using SPSS 18.0 software. The data are expressed as mean ± SD. Difference of parameter between two samples was compared using Student’s *t*-test. Differences among three and more parameters were analyzed by one-way analysis of variance. A value of *P* < 0.05 was considered as statistically significant.

## Results

### Effects of KLT on tumor growth

**Figure 1:**
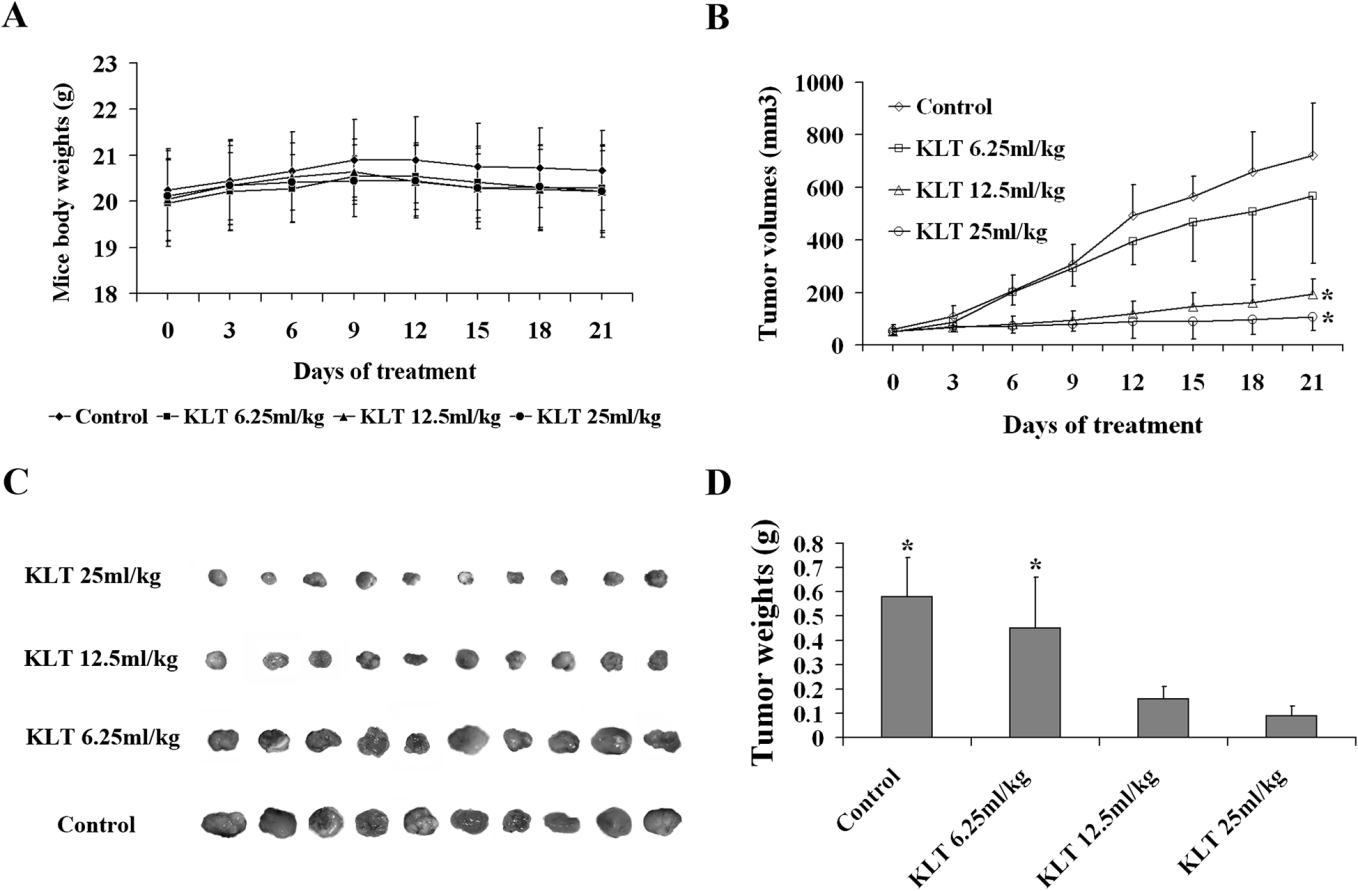
**Effect of KLT on pancreatic cancer xenograft growth.** When the tumors initially formed, the mice were divided into four groups: a control group and a KLT 6.25, 12.5, and 25 ml/kg group. The mice in the KLT-treated groups were intraperitoneally injected daily with KLT for 21 days and the mice in the control group were injected with 0.9% normal saline. **A**. Average body weight of the mice in each group was not significantly different during the treatment (*P* > 0.05). **B**, **C**, and **D**: On the day before KLT treatment, the tumor volumes and weights in the KLT-treated groups were not different compared with the control group (*P* > 0.05). After treatment with KLT for 21 days, the 12.5 and 25 mL/kg KLT-treated groups showed considerably lower tumor volume and weight compared with those in the control group (*P < 0.05).

To determine whether KLT has an anti-tumor effect *in vivo*, a pancreatic carcinoma model was established by subcutaneously injecting PANC-1 cells into nude mice. After treatment with KLT for 21 days, none of the mice exhibited any physical discomfort, and the average body weight of the mice in each group was not significantly different (*P* > 0.05; Figure 1A). On the day before KLT treatment, the tumor volumes were 48.96 ± 11.66 mm^3^ in the KLT 6.25 mL/kg group, 51.47 ± 9.68 mm^3^ in the KLT 12.5 mL/kg group, and 53.44 ± 12.98 mm^3^ in the KLT 25 mL/kg group. Tumor volumes were not different compared with those in the control group (59.16 ± 17.32 mm^3^) (*P* > 0.05; Figure 1B). After treatment with KLT for 21 days, KLT-treated mice had considerably lower tumor volume and weight: 566.01 ± 256.04 mm^3^ (0.45 ± 0.21 g) in the KLT 6.25 ml/kg group, 193.58 ± 57.29 mm^3^ (0.16 ± 0.05 g) in the KLT 12.5 mL/kg group, and 108.18 ± 52.33 mm^3^ (0.09 ± 0.04 g) in the KLT 25 mL/kg group, compared with their previous values. The volumes and weights in the KLT 12.5 mL/kg and 25 mL/kg groups were significantly lower than those of the control group at 720.48 ± 200.28 mm^3^ (0.58 ± 0.16 g; *P* < 0.05; Figure 1B, 1C, and 1D).

### Effect of KLT on tumor cell apoptosis

**Figure 2:**
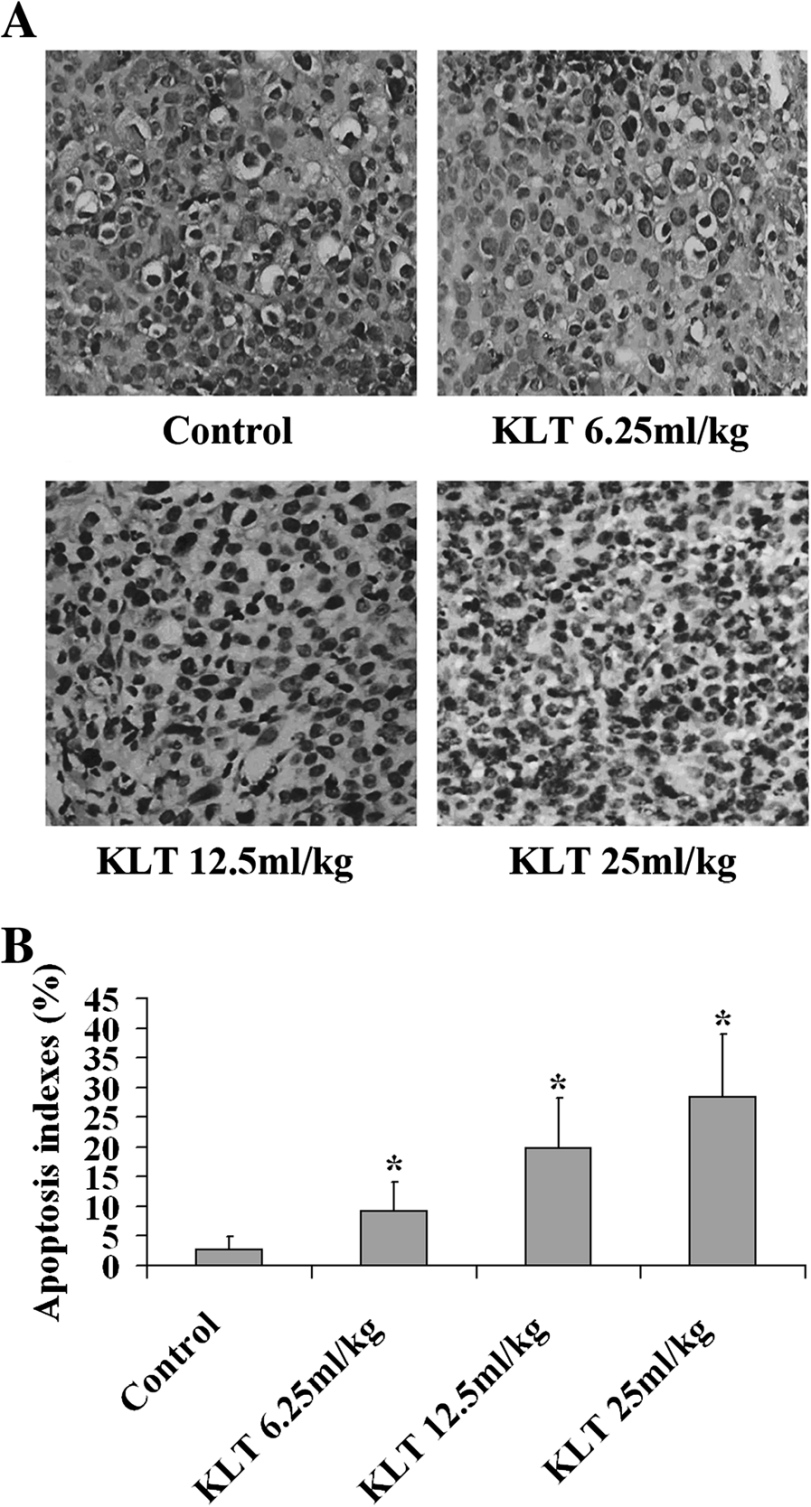
**Effect of KLT on the apoptosis of tumor xenografts. A**. TUNEL assay of tumor xenograft tissue (magnification: ×400). **B**. The apoptosis indexes in KLT-treated groups was significantly higher than those in the control group (*P < 0.05).

To determine whether KLT can effectively induce pancreatic cancer cell apoptosis *in vivo*, tumor sections were analyzed by TUNEL assay. TUNEL assays showed evident *in situ* apoptosis in the 6.25, 12.5, and 25 mg/kg KLT-treated groups (Figure 2A). The apoptosis indexes in the KLT-treated groups were 9.16 ± 4.87%, 19.76 ± 8.44%, and 28.43 ± 10.59%, respectively, and were significantly higher than those in the control group at 2.75 ± 2.14% (*P* < 0.05; Figure 2B).

### Effect of KLT on the expression of components involved in the PI3K/Akt/mTOR pathway

**Figure 3:**
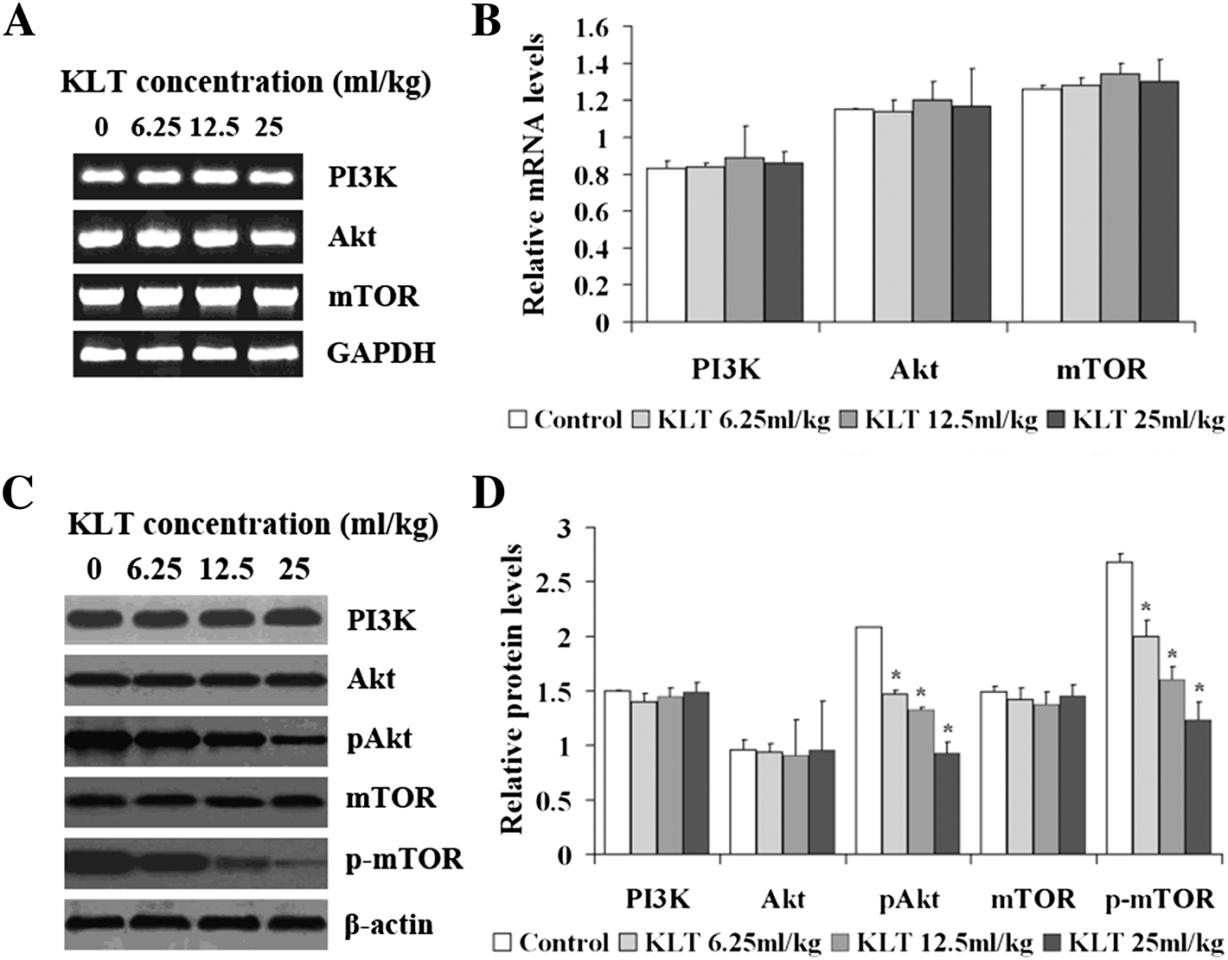
**Effect of KLT on the expression of components involved in the PI3K/Akt/mTOR pathway. A**. After treatment with KLT for 21 days, PI3K, Akt, and mTOR mRNA expression levels of tumor xenografts were examined by RT-PCR, and the PCR products were analyzed by electrophoresis on a 2% agarose gel, followed an ethidium bromide stain. **B**. The expression levels of PI3K, Akt, and mTOR mRNA were unchanged (*P* > 0.05) in the KLT-treated groups compared with the control group after treatment. **C**. After treatment with KLT for 21 days, PI3K, Akt, pAkt, mTOR, and p-mTOR expression levels were detected by western blotting. **D**. After treatment with KLT for 21 days, the pAkt and p-mTOR expression levels were significantly lower than those in the control group (*P < 0.05). The expression levels of PI3K, Akt, and mTOR were the same as those in the control group (*P* > 0.05).

The modulating effect of KLT on PI3K, Akt, and mTOR mRNA expression is shown in Figure 3A and 3B. GAPDH mRNA expression levels in KLT-treated groups were no different than those in the control group (*P* > 0.05). The expression levels of PI3K, Akt, and mTOR mRNA were no different (*P* > 0.05) in the KLT-treated groups compared with those in the control group after the mice were treated with KLT for 21 days.

Next, we investigated the modulating effect of KLT on the expression levels of PI3K, Akt, p-Akt, mTOR, and p-mTOR (Figure 3C, 3D). After the mice were treated with different concentrations of KLT for 21 days, the expression levels of β-actin in the KLT-treated groups were no different than those in the control group (*P* > 0.05). Expression levels of pAkt and p-mTOR were significantly lower than those in the control group after treatment (*P* < 0.05). The expression levels of PI3K, Akt, and mTOR were no different than those in the control group (*P* > 0.05).

## Discussion

Our study was performed in nude mice bearing subcutaneous PANC-1 human pancreatic cancer xenografts. The regression of tumor implants was measured in all KLT-treated animals, and no overtly toxic effects or effects on weight gain were observed. Furthermore, decreased expression levels of pAkt and p-mTOR were detected in KLT-treated tumors.

KLT has an inhibitory effect on many types of tumors [7]. In our study, after treatment with KLT for 21 days, the tumor volumes in KLT-treated groups were considerably lower than those in the control group (*P* < 0.05). The tumor inhibition ratios in the 25 and 12.5 mL/kg groups were 84.48% and 72.41%, respectively, while the 6.25 mL/kg group was only 22.41% (*P* < 0.05). These data illustrate that KLT contributed to the inhibition of tumor growth in the xenograft animal model, which confirm the findings of other researchers. Wu *et al.* found that growth inhibition ratios in hepatoma xenografts were 46.4% and 49.4% after treatment with 5% and 10% KLT for 8 days [6]. Moreover, Pan *et al.* reported that the inhibition ratios were 33.25% and 43.89% after lung cancer xenografts were treated with 6.25 and 12.5 mL/kg of KLT for 14 days [3]. Discrepancies in the experimental methods used in these studies could explain the diversities among these findings. The injection doses used in this study are similar to those in other studies. The equivalent dose of 25 mL/kg to a human being is 2.77 mL/kg. The clinical dosage of KLT used in China is 200 mL/person (70 kg), which is approximately 2.86 mL/kg.

Clinical applications show that KLT can improve immune function, reduce chemotherapy toxicity [5], and relieve cancer pain, thus improving the quality of life of patients [2]. In the present study, mice treated with KLT for 21 days showed no symptoms of physical discomfort, and no significant differences were observed in average body weight of the mice among the groups (*P* > 0.05). This suggests that KLT did not produce significant non-tumor toxicity in tumor-bearing mice.

Cell and animal experiments show that KLT can activate some pro-apoptotic factors and block the tumor cell cycle, further leading to tumor cell apoptosis [7,8]. TUNEL assays showed that the apoptosis indexes in the KLT-treated groups were 9.16 ± 4.87%, 19.76 ± 8.44%, and 28.43 ± 10.59%, for 6.25, 12.5, and 25 mg/kg, respectively. These indexes were significantly higher than those in the control group (*P* < 0.05). Therefore, KLT administration inhibited PANC-1 xenograft tumor growth via induction of tumor cell apoptosis. Similarly, Ye *et al.* found that the apoptosis index was 55 ± 8% after liver cancer xenografts were treated with KLT for 15 days [17].

The PI3K/Akt/mTOR pathway is very important in the development of cancers, and plays a key role in cellular functions including metabolism, survival, proliferation, adhesion, migration, and invasion [18]. Phosphatidylinositol3-kinases are a family of enzymes that phosphorylate phosphatidylinositol. Phosphatidylinositol 3,4,5-triphosphate is a messenger generated by PI3K, which activates Akt. Akt controls protein synthesis and cell growth by the phosphorylation of mTOR [18]. In the present study, the expression levels of PI3K, Akt, and mTOR mRNA and protein were unchanged (*P* > 0.05) in KLT-treated groups compared with those in the control group. However, pAkt and p-mTOR expression levels were significantly lower than those in the control group (*P* < 0.05). Therefore, KLT can inhibit PI3K/Akt/mTOR signaling via inhibiting Akt and mTOR phosphoration, rather than by influencing Akt and mTOR.

## Conclusions

KLT can inhibit proliferation and induce apoptosis in human pancreatic cancer xenografts. In addition, KLT can downregulate pAkt and p-mTOR expression to regulate PI3K/Akt/mTOR signaling, which may contribute to the anti-proliferative and apoptosis-inducing properties of KLT. Nevertheless, further investigation is essential to reveal the modulating effect of KLT on proteins downstream of the PI3K/Akt/mTOR pathway.

## Competing interests

The authors declare that they have no competing interests.

## Authors’ contributions

LS and LY designed the research; LY and WXJ contributed to the animal experiments; LY and ZW performed all the assays; LY and ZW analyzed the data; LY, ZW, and LS wrote the paper. All authors have read and approved the final manuscript.

## Pre-publication history

The pre-publication history for this paper can be accessed here:

http://www.biomedcentral.com/1472-6882/14/228/prepub
